# Propofol‐alone sedative efficacy in observational biliopancreatic endoscopic ultrasound

**DOI:** 10.1002/deo2.70025

**Published:** 2024-10-17

**Authors:** Hisaki Kato, Yuki Kawasaki, Kazuya Sumi, Yuki Shibata, Norihiro Nomura, Jun Ushio, Junichi Eguchi, Takayoshi Ito, Haruhiro Inoue

**Affiliations:** ^1^ Department of Digestive Diseases Center Showa University Koto Toyosu Hospital Tokyo Japan

**Keywords:** analgesics, endoscopic ultrasonography, propofol, sedative, sedative efficacy

## Abstract

**Objectives:**

Appropriate sedative and analgesic selection is essential to reduce patient discomfort and body movement to safely conduct endoscopic ultrasonography (EUS). However, few cases have examined sedation with propofol in EUS, and few studies the need for combined analgesia. In this study, we retrospectively evaluated the usefulness and safety of propofol without analgesics for sedation in biliopancreatic observational EUS.

**Methods:**

This single‐center retrospective study included 516 observational biliopancreatic EUS procedures using propofol alone performed between April 2021 and March 2023. The primary and secondary endpoints were the observational biliopancreatic EUS results obtained with propofol alone and adverse event occurrence, respectively.

**Results:**

The median examination time and total propofol dose were 22 (range: 10–67) min and 186.5 (range: 50–501) mg, respectively. The median starting Richmond Agitation‐Sedation Scale and Visual Analog Scale scores were −5 (range: −5–1) and 0 (range: 0–10), respectively. The median recovery time was 22 (range: 5–80) min. Adverse events occurred in 60 (11.6%) patients. Trainee‐performed examination (odds ratio [OR] 3.52, 95% confidence interval [CI]: 1.63–7.60, *p* = 0.0014) and examination length (>22 min; OR 1.67, 95% CI: 0.95–2.92, *p* = 0.07) were risk factors for adverse events.

High body mass index (OR 1.87, 95% CI: 1.10–3.16, *p* = 0.02) and extended examination time (OR 4.23, 95% CI: 2.08–8. 57, *p* < 0.001) were risk factors for delayed recovery.

**Conclusions:**

During observational biliopancreatic EUS, propofol is useful as a single sedative and offers high patient satisfaction and relative safety.

## INTRODUCTION

Sedation endoscopy is preferred for its focus on safety and high patient satisfaction. It has been reported to be beneficial for both patients and endoscopists in improving the quality of examinations.^1^, ^2^

In recent years, endoscopic ultrasonography (EUS) has become increasingly important in the rising incidence of biliopancreatic diseases and early diagnosis of biliopancreatic cancer because of its high visualization ability.^3^

Endoscopic retrograde cholangiopancreatography (ERCP) and EUS have a larger scope diameter and longer examination time compared to upper gastrointestinal endoscopy. Therefore, it is crucial to choose proper sedatives and analgesics to minimize patient discomfort and movement for safe ERCP and EUS procedures.

Common hypnotic sedatives include benzodiazepines, dexmedetomidine hydrochloride, pethidine hydrochloride, and fentanyl. Pentazocine acts as an antagonistic analgesic. Propofol is an ultrashort‐acting sedative given intravenously, which sedates patients rapidly and effectively. Unlike opioids, it lacks analgesic properties but has a shorter elimination half‐life and quicker arousal time. Additionally, it is associated with fewer adverse events like nausea and vomiting.

Reportedly, propofol usage in endoscopy results in shorter recovery times, fewer interruptions during long procedures, and increased satisfaction among physicians, nurses, and patients.^4^

A study on sedation related to ERCP revealed that a randomized controlled trial comparing sedatives alone and sedatives plus analgesics groups reported reduced pain and higher satisfaction levels among patients and endoscopists in the combination group.^5^ Additionally, a meta‐analysis of randomized controlled trials comparing propofol use in ERCP and EUS with conventional sedatives showed no significant differences in the incidence of hypotension, hypoxemia, or procedure time, but did reveal a significantly quicker recovery time.^6^, ^7^ Yusoff et al. also reported that during upper gastrointestinal EUS examinations using propofol, all patients preferred the same sedation for their subsequent examination.^8^

Various comparative studies have been conducted on sedation and analgesia in ERCP. However, none have compared the use of sedatives alone with the use of both sedatives and analgesics in EUS. Additionally, the use of sedation with propofol during EUS has only been studied in a limited number of cases, with few studies looking into the necessity of combined analgesia.^9^, ^10^

In this study, we evaluated the usefulness and safety of propofol sedation without analgesics during biliopancreatic observational EUS.

## MATERIALS AND METHODS

### Patients

**FIGURE 1:**
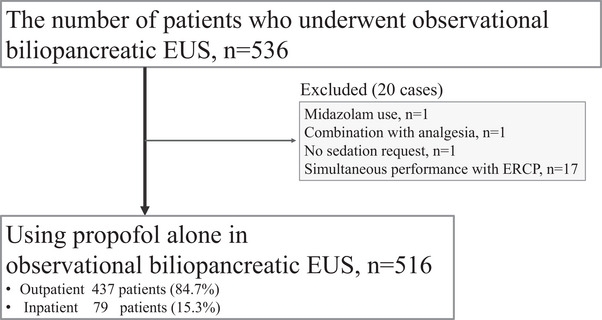
The selection of patients who underwent observational biliopancreatic endoscopic ultrasonography (EUS).

This is a single‐center retrospective analysis of patients sedated with propofol only and without analgesics during biliopancreatic EUS observation. We included observational biliopancreatic EUS performed between April 2021 and March 2023. The selection process is illustrated in Figure 1. During the study period, 536 patients underwent biliopancreatic EUS observation. Of these, 20 patients (3.7%) were excluded (one patient using midazolam, one patient with analgesia, one patient requesting no sedation, and 17 patients who underwent simultaneous ERCP). We retrospectively examined 516 patients (96.2%).

### Observational EUS

Observational biliopancreatic EUS was performed using an echoendoscope (GF‐UE290 and GF‐UCT260; Olympus Medical Systems) and an endoscopic ultrasound observation device (EU‐ME2; Olympus Medical Systems).

All patients were checked for medical history, risk assessment by comorbidity (cardiac disease [ischemia and arrhythmia], chronic obstructive pulmonary disease [COPD], and sleep apnea syndrome [SAS]), medications, and allergies. Pre‐sedation evaluation was performed using a checklist.

The trainee and/or trainer performed observation EUS, and all patients were sedated with propofol alone. All patients were anesthetized by the EUS physician or trainer. Even when the trainee performed EUS, it was supervised by an endoscopist (trainer) with at least 5 years of EUS experience and at least 100 cases of EUS‐guided tissue acquisition (EUS‐TA), who also made the final diagnosis.

Sedation was initiated using propofol at a dose of 0.5–1.0 mg/kg for induction, followed by a continuous maintenance dose of 3 mg/kg/h administered via a syringe pump.

Additional propofol in 10–20‐mg increments was given as needed during movement or poor sedation, with repeat doses administered until adequate anesthesia induction was achieved. Bolus and sustained doses of propofol at induction, as well as reasons for any extra bolus (such as body movement or pain), were recorded during the examination. Vital signs were monitored every 5 min for all patients during the procedure, with monitoring every 10 min in the recovery room afterward. Symptoms such as abdominal pain, nausea, and recovery time were also noted. Patient satisfaction was assessed pre‐discharge using a visual analog scale (VAS).

All patient records were collected and documented in a checklist format by the nurses at the endoscopy center.

### Outcome measures

The primary endpoint of the study was the observational biliopancreatic EUS results using propofol alone. This included the patient's distress levels, adverse events, and recovery time from completion of examination to release from rest in outpatients.

The secondary endpoints were adverse event factors, propofol dose, and examination time.

### Definition

The Richmond Agitation‐Sedation Scale (RASS) score was expressed as positive and negative for arousal and sedation, respectively, with +4 for dangerously excited, 0 for calm, and −5 for unarousable.^11^, ^12^

Patient satisfaction was measured using the VAS, with 0 indicating comfort and 10 indicating discomfort.

Respiratory depression was defined as pulse oxygen saturation (SpO_2_) <90%, necessitating oxygen administration.

Hypotension was defined as systolic blood pressure <90 mmHg and bradycardia as heart rate <50 bpm.

The anesthesia recovery score determined when a patient could be released from rest, assessed by levels of consciousness, motor function, respiration, circulation, and SpO_2_ (Table 1).^13^, ^14^, ^15^ An anesthesia recovery score of 8 or higher allowed rest release.

**TABLE 1:** Anesthesia recovery score.

Criteria	Score
Activity	
Movement, spontaneously or on command	2
Weak movement, spontaneously or on command	1
No movement	0
Respiration	
Coughs on command or cries	2
Maintains airway without support	1
Airway maintenance required	0
Systolic blood pressure	
±20 mmHg of pre‐anesthetic level	2
±20–50 mmHg of pre‐anesthetic level	1
>50 mmHg of pre‐anesthetic level	0
Consciousness	
Wakefulness or easily awakened when called	2
Defensive reflexes to stimuli	1
No response or absence of defensive reflexes	0
O_2_ saturation	
Saturation ≥92% or ≥ pre‐anesthetic value in room air	2
Saturation ≥92% or ≥ pre‐anesthetic value with	1
supplemental O_2_	
Saturation ≤92%	0

The time from the end of the examination until the patient was released from rest and could return home was the recovery time, with delayed recovery time being at least 30 min.

Adverse events were defined based on the lexicon of endoscopic adverse events as outlined by the American Society for Gastrointestinal Endoscopy.^16^

Gastroenterologists who were able to complete routine abdominal ultrasonography and upper and lower endoscopy independently were assigned as trainees to begin training. A trainer must have at least 5 years of EUS experience and at least 100 cases of EUS‐TA at a specialized institution.

### Statistical analysis

Continuous variables, including age and length of the procedure, were presented as medians and ranges, and categorical variables as proportions. The univariate analyses were performed using the χ^2^ or Fisher's exact test for categorical variables and the Mann–Whitney U‐test for continuous variables. Statistical significance was set at *p* < 0.05. Multivariate analysis was performed using logistic regression modeling, and factors with a univariate *p*‐value of <0.05 were entered into the multivariate model. All data analyses were performed using the JMP Statistics for Windows (version 17.0; SAS Institute Japan Corp.).

### Ethics approval

The Showa University Koto Toyosu Hospital Review Board (No. 2023‐229‐B) approved this study. This study conformed to the provisions of the Declaration of Helsinki (revised in Fortaleza, Brazil, October 2013).

## RESULTS

### Patient background

The clinical characteristics of the 516 patients (254 [49.2%] males and 262 [50.8%] females) are presented in Table 2. The patients’ median age and body mass index (BMI) were 65 (range: 25–94) years and 22.4 (range: 14.0–38.1) kg/m^2^, respectively. Outpatient procedures were performed in 437 patients (84.7%) and inpatient procedures in 79 patients (15.3%). In total, 113 (21.9%) patients underwent esophagogastroduodenoscopy (EGD) simultaneously with observational biliopancreatic EUS. The radial type was used in 510 cases (98.8%) and the convex type in six cases (1.2%). Trainees, alongside their trainers, performed 343 (66.5%) cases, while 173 (33.5%) were performed by trainers alone. Of these, 43 patients (8.3%) had comorbidities (one [0.2%] with SAS, three [0.6%] with COPD, and 39 [7.5%] with cardiac disease).

**TABLE 2:** Clinical characteristics of the study population.

Variables	*n* = 516
Median age, years (range)^a^	65 (25–94)
Male sex, *n* (%)	254 (49.2)
Median height, m (range)	1.61 (1.35–1.9)
Median body weight, kg (range)	57.7 (34.2–131.8)
Median BMI, kg/m^2^ (range)	22.4 (14.0–38.1)
Outpatient, *n* (%)	437(84.7)
Simultaneous performance of EGD, *n* (%)	113 (21.9)
Scope type of EUS, *n* (%)	
Radial	510 (98.8)
Convex	6 (1.2)
EUS provider, *n* (%)	
Trainee + trainer	343 (66.5)
Trainer	173 (33.5)
Comorbidity, *n* (%)	43 (8.3)
SAS	1 (0.2)
COPD	3 (0.6)
Cardiac disease (ischemia and arrhythmia)	39 (7.5)

Abbreviations: BMI, body mass index; COPD, chronic obstructive pulmonary disease; EGDS, esophagogastroduodenoscopy; EUS, endoscopic ultrasonography; SAS, sleep apnea syndrome.

^a^ Age at which EUS was performed.

### Sedation results of observational biliopancreatic EUS

The median examination time was 22 (range: 10–67) min. The median total propofol dose was 186.5 (range: 50–501) mg. The median starting RASS score was −5 (range: −5–1), and the median VAS score was 0 (range: 0–10). The median recovery time was 22 (range: 5–80) min. Adverse events were observed in 60 patients (11.6%), including respiratory depression in 25 (4.8%), hypotension in 15 (2.9%), bradycardia in six (1.2%), disinhibition in 12 (2.3%), extravascular propofol leakage in one (0.2%), and acute pancreatitis in one patient (0.2%; Table 3). All the adverse events were of mild grade. No re‐sedation was observed after the patient returned home. All the adverse events overlapped with respiratory depression: nine cases of hypotension, one case of bradycardia, and four cases of de‐suppression.

**TABLE 3:** Endoscopic ultrasonography results for the study population.

Variables	*n* = 516
Median examination time, min (range)	22 (10–67)
Median total propofol dose, mg (range)	186.5 (50–501)
Median starting RASS, points (range)	−5 (−5–1)
Median VAS, points (range)	0 (0–10)
Median time to awaken, min (range)	22 (5–80)
Adverse events, *n* (%)	
Hypoxia	25 (4.8)
Hypotension	15 (2.9)
Bradycardia	6 (1.2)
Disinhibition	12 (2.3)
Propofol leakage	1 (0.2)
Acute pancreatitis	1 (0.2)

Abbreviations: RASS, Richmond agitation‐sedation scale; VAS, visual analog scale.

### Clinical characteristics and EUS results of adverse events

Adverse event factors analyzed were age, sex, BMI, simultaneous performance of EGD, EUS provider (performed by a trainee), comorbidities (SAS, COPD, and cardiac disease), examination time, and total propofol dosage. The risk of adverse events was significantly associated with three factors: trainee‐performed tests (*p* = 0.0007), COPD comorbidities (*p* < 0.001), and high propofol dosage (*p* = 0.04; Table 4). In addition, univariate and multivariate analyses were performed to examine factors contributing to adverse events with respect to age (>65 years, ≤65 years), sex, BMI (>25 kg/m^2^, ≤25 kg/m^2^), whether EGD was performed simultaneously, who performed the EUS (Trainee and Trainer or Trainer alone), presence of comorbidity, examination time (>22 min, ≤22 min), and total propofol dosage (≥180 mg, <180 mg; Table 5). The groups were divided based on the median age, examination time, and propofol dose. In the univariate analysis, the examination performed by the trainee (odds ratio [OR] 3.52, (95% confidence interval [CI]: 1.63–7.60, *p* = 0.0014) was considered the risk factor for adverse events.

**TABLE 4:** Clinical characteristics and endoscopic ultrasonography (EUS) results of adverse events.

Variables	Total *n* = 516	Without adverse events *n* = 457	With adverse events *n* = 59	*p*‐value
Median age, years (range)	65 (25–94)	65 (25–94)	65 (31–92)	0.68
Male sex, *n* (%)	254 (49.2)	227 (49.7)	27 (45.8)	
Median BMI, kg/m^2^ (range)	22.4 (14.0–38.1)	22.4 (14.0–38.1)	22.4 (14.2–30.8)	0.8
Simultaneous performance of EGD, *n* (%)	113 (21.9)	103 (22.5)	10 (16.9)	0.36
EUS provider, *n* (%)				
Trainee + trainer	343 (66.5)	293 (64.1)	50 (84.7)	0.0007
Comorbidity, *n* (%)	43 (8.3)	35 (7.6)	8 (13.6)	0.11
SAS	1 (0.2)	1 (0.2)	0 (0)	0.72
COPD	3 (0.6)	1 (0.2)	2 (3.4)	<0.001
Cardiac disease	39 (7.5)	33 (7.2)	6 (10.2)	0.3951
Median examination time, min (range)	22 (10–67)	21 (10–67)	25 (10–53)	0.11
Median total propofol dose, mg (range)	186.5 (50–501)	180 (50–501)	210 (50–490)	0.04

Abbreviations: BMI, body mass index; COPD, chronic obstructive pulmonary disease; EGDS, esophagogastroduodenoscopy; EUS, endoscopic ultrasonography; SAS, sleep apnea syndrome.

**TABLE 5:** Factors affecting the risk of adverse events during observational endoscopic ultrasonography.

			**Univariate analysis**			**Multivariate analysis**		
**Variable**	***n***	**Adverse event risk (%)**	**OR**	**95% CI**	***p*‐value**	**OR**	**95% CI**	***p*‐value**
Age, years								
<65	250	11.2 (28/250)						
≥65	266	11.7 (31/266)	1.1	0.64–1.91	0.74	‐	‐	‐
Sex								
Male	254	10.6 (27/254)						
Female	262	12.2 (32/262)	1.13	0.65–1.95	0.67	‐	‐	‐
BMI								
<25	404	10.4 (42/404)						
≥25	112	15.2 (17/112)	1.42	0.76–2.63	0.27	‐	‐	‐
Simultaneous performance of EGD								
Yes	113	8.8 (10/113)						
No	403	12.2 (49/403)	1.39	0.68–2.85	0.36	‐	‐	‐
Provider of EUS								
Trainer alone	173	5.2 (9/173)						
Trainer + trainee	343	14.6 (50/343)	3.52	1.63–7.60	0.0014	‐	‐	‐
Comorbidity								
No	473	10.8 (51/473)						
Yes	43	18.6 (8/43)	1.93	0.85–4.40	0.12	‐	‐	‐
Examination time (min)								
<22	253	8.7 (22/253)						
≥22	263	14.1 (37/263)	1.67	0.95–2.92	0.07	‐	‐	‐
Total propofol dose (mg)								
>180	262	13.7 (36/262)						
≤180	254	9.1 (23/254)	1.57	0.88–2.78	0.12	‐	‐	‐

Abbreviations: BMI, body mass index; EGDS, esophagogastroduodenoscopy; EUS, endoscopic ultrasonography.

### Factors affecting delayed recovery time in observational EUS

Recovery time was considered only in outpatients based on whether the patient could return home.

Factors that delayed recovery time included age, sex, BMI, simultaneous EGD, EUS provider (performed by a trainee), comorbidities (SAS, COPD, and cardiac disease), examination time, total propofol dosage, and adverse events.

Only the BMI (*p* = 0.049) was a significant risk factor for delayed recovery time (Table 6).

**TABLE 6:** Comparison of clinical characteristics of delayed recovery time on observational endoscopic ultrasonography.

Variables	Total *n* = 437	Recovery time <30 *n* = 327	Recovery time ≥30 *n* = 110	*p*‐value
Median age, years (range)	64 (28–90)	63 (30–90)	65 (30–89)	0.58
Male sex, *n* (%)	219 (50.1)	169 (51.6)	50 (45.5)	
Median BMI, kg/m^2^ (range)	22.4 (14.0–38.1)	22.6 (14.0–38.1)	22.0 (14.0–31.2)	0.049
Simultaneous performance of EGD, *n* (%)	88 (20.1)	61 (18.7)	27 (24.5)	0.29
EUS provider, *n* (%)				
Trainee + trainer	286 (65.4)	213 (65.1)	73 (66.4)	0.82
Comorbidity, *n* (%)	32 (7.3)	21 (6.4)	11 (10.0)	0.21
SAS	1 (0.2)	1 (0.3)	0 (0)	0.56
COPD	1 (0.2)	0 (0)	1 (0.9)	1.00
Cardiac disease	31 (7.1)	21 (6.4)	10 (9.1)	0.29
Median examination time, min (range)	21 (10–67)	21 (10–67)	22 (10–51)	0.62
Median total propofol dose, mg (range)	180 (60–490)	180 (60–490)	180 (80–450)	0.66
Adverse events, *n* (%)	51 (11.7)	36 (11.0)	15 (13.6)	0.46

Abbreviations: BMI, body mass index; COPD, chronic obstructive pulmonary disease; EGDS, esophagogastroduodenoscopy; EUS, endoscopic ultrasonography; SAS, sleep apnea syndrome.

In addition, univariate and multivariate analyses were performed for age (>65 years, ≤65 years), sex, BMI (>25 kg/m^2^, ≤25 kg/m^2^), whether EGD was performed simultaneously, adverse events, comorbidities, examination time (>22 min, ≤22 min), and total propofol dose (≥180 mg, <180 mg; Table 7). The groups were divided based on the median age, examination time, and propofol dose. In univariate analysis, high BMI (OR 1.72 [95% CI: 1.05–2.82, *p* = 0.03]), simultaneous performance of EGD (OR 1.74 [95% CI: 1.07–2.82, *p* = 0.03]), long examination time (>22 min; OR 1.64 [95% CI: 1.05–2.56, *p* = 0.03]) were risk factors for delayed recovery. In multivariate analysis, high BMI (OR 1.87 [95% CI: 1.10–3.16, *p* = 0.02]), simultaneous performance of EGD (OR 1.68 [95% CI: 0.97–2.92, *p* = 0.06]), long examination time (>22 min; OR 4.23 [95% CI: 2.08–8. 57, *p* < 0.001]), indicating that a high BMI and long examination time were risk factors for delayed recovery.

**TABLE 7:** Factors affecting delayed recovery time in observational endoscopic ultrasonography.

			Univariate analysis			Multivariate analysis		
Variable	*n*	Recovery time ≥30 (%)	**OR**	**95% CI**	***p*‐value**	**OR**	**95% CI**	***p*‐value**
Age, years								
<65	229	21.4 (49/229)						
≥65	208	29.3 (61/208)	1.08	0.7–1.67	0.72	‐	‐	‐
Sex								
Female	218	23.9 (52/218)						
Male	219	26.5 (58/219)	1.18	0.76–1.82	0.46	‐	‐	‐
BMI								
<25	339	23.0 (78/339)						
≥25	98	32.7 (32/98)	1.72	1.05–2.82	0.03	1.87	1.10–3.16	0.02
Simultaneous performance of EGD								
No	349	21.8 (76/349)						
Yes	88	38.6 (34/88)	1.74	1.07–2.82	0.03	1.68	0.97–2.92	0.06
Adverse events								
Yes	51	13.7 (7/51)						
No	386	26.6 (103/386)	1.47	0.71–3.05	0.3	‐	‐	‐
Comorbidity								
No	405	23.5 (95/405)						
Yes	32	46.9 (15/32)	1.99	1.00–3.94	0.05	‐	‐	‐
Examination time (min)								
<22	228	9.6 (22/228)						
≥22	209	17.7 (37/209)	1.64	1.05–2.56	0.03	4.23	2.08–8.57	<0.001
Total propofol dose (mg)								
>180	216	16.7 (36/216)						
≤180	221	10.4 (23/221)	1.24	0.80–1.93	0.34	‐	‐	‐

Abbreviations: BMI, body mass index; COPD, chronic obstructive pulmonary disease; EGDS, esophagogastroduodenoscopy; SAS, sleep apnea syndrome.

## DISCUSSION

In this study, we found that using propofol alone for observational biliopancreatic EUS led to increased patient satisfaction, fewer adverse events, and quicker recovery time post‐examination before discharge.

Liang et al. compared propofol‐dexmedetomidine and propofol‐remifentanil during EUS examination and reported mean total doses of 233 and 244 mg, respectively.^10^ Okamoto et al. reported a median total dose of 159 mg for propofol sedation during biliopancreatic EUS using a Bispectral Index (BIS) monitor without mention of concomitant analgesia.^17^ The median total propofol dose at our hospital was 186.5 mg, and there was no significant difference in total propofol dose between analgesic use and no analgesic use compared to previous reports.

Regarding examination time and patient satisfaction, Liang et al. reported a mean time of 27.3 min and a mean VAS score of 0.12.^10^ In this study, the median examination time was 22 min, and although there was a difference between the mean and median, the examination time did not differ significantly from that in previous reports of concomitant analgesia.

Furthermore, the median VAS score was 0, indicating high patient satisfaction with or without the use of analgesics. The previous study did not mention the performing physician; however, this present study showed no significant difference in VAS scores and a high level of satisfaction, even if the performing physician was a trainee.

Liang et al. and Okamoto et al. reported adverse events in 10.2% and 17.3% of patients, respectively.^10^, ^17^ Yusoff et al. reported mild hypoxemia in 20% of cases but no cases of severe hypoxemia (saturation <85%).^8^ In this study, the rate of adverse events was 11.6%, which could be improved through appropriate monitoring.

Regarding the risk of adverse events, a significant difference was observed in the risk of adverse events due to COPD comorbidity; however, the number of patients was only 3, and further studies are warranted, including more cases. Moreover, a significant difference was observed in examinations conducted solely by trainees. This is assumed to be because of their tendency to cause patient movement through excessive endoscopic maneuvering. As a result, localized propofol administration may be necessary, leading to potential adverse events. Trainers should appropriately control the examination time and endoscopic maneuvering of the trainees.

In outpatients, Liang et al. reported a mean time of 16.56 ± 3.91 and 15.39 ± 3.8 min from the end of the examination to the release from rest for the propofol‐dexmedetomidine and propofil‐remifentanil groups, respectively.^10^ Okamoto et al. also reported that propofol sedation during EUS examinations using a BIS monitor resulted in a recovery time of 22.5 min.^17^ Despite the difference in the method used to evaluate the exit criteria, the median recovery time in this study was 22 min and no differences were found.

Significant differences were found between high BMI and long examination times as risk factors for delayed recovery time. This may be because obesity can affect propofol blood concentration, making it challenging to adjust the effective threshold at high BMI. Drug doses may also be excessive based on total body weight.^18^, ^19^

In addition, acute pancreatitis was observed in one patient (0.002%) as a post‐examination adverse event. In this case, no stressful endoscopic manipulation, such as balloon dilation at the papillary site, was performed. Therefore, we considered this case to be drug‐induced pancreatitis caused by propofol. The clinical course of the patient improved quickly after fasting and supplemental fluid therapy alone.

Notably, several case reports have indicated an association between propofol and pancreatitis.^20^, ^21^ The exact mechanisms are still unclear, but proposed causes include hypertriglyceridemia, hypersensitivity, and direct pancreatic toxicity of the drug.^22^ It is essential to consider acute pancreatitis as an adverse event of propofol.

In surgery, sedation involves using a combination of drugs with varying mechanisms of action to minimize individual side effects.^23^, ^24^ However, in this study, sedation with propofol alone in observational EUS showed no difference in patient satisfaction, adverse events, or patient recovery time compared with previous reports.

This study has limitations as it was a single‐center, retrospective study without a comparison of analgesic combinations and used a biased echoendoscope. Therefore, a multicenter, prospective comparative study with a combined analgesic group is recommended to eliminate bias from the echoendoscope.

Trainers should appropriately control the examination time and endoscopic maneuvering of the trainees. However, the number of additional doses was not examined in this study, and a comparison of the number of local doses by the trainee and trainer is considered necessary in the future.

In conclusion, propofol alone is an effective sedation method for observational biliopancreatic EUS, resulting in high patient satisfaction and overall safety.

## CONFLICT OF INTEREST STATEMENT

Haruhiro Inoue, who received grants from the Olympus Corporation and Takeda Pharmaceutical Company, is an advisor for Olympus Corporation and Top Corporation. The other authors declare no conflict of interest.

## ETHICS STATEMENT

The Showa University Koto Toyosu Hospital Review Board (No. 2023‐229‐B) approved this study. This study conformed to the provisions of the Declaration of Helsinki (revised in Fortaleza, Brazil, October 2013).

## PATIENT INFORMED CONSENT

With approval from the Showa University Koto Toyosu Hospital Review Board (No. 2023‐229‐B), informed consent was obtained through an opt‐out method. In this method, instead of obtaining written consent from each individual, the research content was disclosed on a web homepage, and the opportunity to refuse participation in the research was provided.  
